# Molecular and Serological Identification of Pathogenic *Leptospira* in Local and Imported Cattle from Lebanon

**DOI:** 10.1155/2023/3784416

**Published:** 2023-02-27

**Authors:** Elena Harran, Alain Abi Rizk, Sophie Angelloz-Pessey, Karine Groud, Virginie Lattard, Christo Hilan, Florence Ayral

**Affiliations:** ^1^VetAgro Sup, Université Claude Bernard Lyon 1, USC 1233, Marcy L'Etoile, Lyon, France; ^2^Faculty of Arts and Sciences, Holy Spirit University of Kaslik (USEK), P.O. Box 446, Jounieh, Lebanon; ^3^VetAgro Sup, Université Claude Bernard Lyon 1, Laboratoire des Leptospires et d'Analyses Vétérinaires, Marcy L'Etoile, Lyon, France

## Abstract

Leptospirosis is a worldwide bacterial zoonosis for which the risk increases in warm and wet climates. Despite the suitability of the local climate for the bacteria's persistence, Lebanon lacks a formal system of prophylaxis for the prevention of *Leptospira* infection in both humans and animals, and the disease's epidemiology is unknown so far. As a preliminary step, we focused on *Leptospira* infection in cattle, which is of public health and economic concern. We conducted a descriptive study in cattle from the governorate of Mount Lebanon (ML) and in imported cattle. A total of 187 blood and 135 serum samples were provided. Among the 187 blood samples, 135 were from randomly selected animals from 14 herds, while the remaining 52 were from imported livestock. Serum specimens (*n* = 135) were obtained exclusively from cattle in the ML governorate. DNA was extracted from all blood samples and subjected to real-time PCR targeting 16S rRNA. All *Leptospira*-positive DNA samples were then amplified using conventional PCR (cPCR), and *Leptospira* species were identified via Sanger sequencing. A microscopic agglutination test (MAT) was performed on the 135 serum samples from local cattle. The real-time PCR revealed *Leptospira* infection in 7 of 135 local animals and 1 of 52 imported animals. DNA from six of the seven local animals and the one imported animal were amplified by cPCR and successfully sequenced, identifying the pathogen as the species *L. kirschneri*. Seven animals located in five out of the 14 tested herds had MAT titers ≥1 : 100. Serogroup Grippotyphosa was predominant. This is the first study to provide epidemiological data on bovine *Leptospira* infection in Lebanon. Pathogenic *Leptospira* species and serogroups were identified in both local and imported cattle. These findings highlight the cattle associated risk of *Leptospira* infection in Lebanon, in the contexts of farming and trade. *Summary*. Leptospirosis is a severe zoonotic disease that can have critical consequences for people and animals. Within the country of Lebanon, this disease has been reported, but its epidemiology is unknown so far. The present study thus provides the first description of the existence of *Leptospira* in cattle in Lebanon (local and imported). It also highlights the existence of different pathogenic serogroups of *Leptospira* in local cattle. Our results should raise public health awareness of the threat posed by this underdiagnosed disease and serve as a starting point for control efforts in Lebanon.

## 1. Introduction

Worldwide, leptospirosis is an important emerging zoonosis caused by pathogenic spirochaetes in the genus *Leptospira* [1]. Tropical and subtropical regions are the most vulnerable to infection [2]. In Lebanon, annual morbidity (i.e., disease incidence) and mortality in humans due to leptospirosis have been estimated at 2.93 IC_95%_ [0.92–5.37] and 0.15 IC_95%_ [0.05–0.26] per 100,000 individuals, respectively, based on age- and gender-adjusted demographic attributes of the human population [3]. However, the epidemiology of leptospirosis in this country remains unknown; no case reports have been published, with the sole exception of a 1947 inquiry launched following the discovery of two cases of Weil's disease [4]. Lebanon lacks a formal system of prophylaxis for leptospirosis despite the suitability of its climate (warm and wet) for the bacteria's persistence and the presence of potential maintenance hosts such as rodents [5, 6]. Furthermore, several domesticated animals that are known to be susceptible to leptospirosis, primarily local and imported cattle (*Bos taurus taurus*), are raised in abundance [7], but their *Leptospira* infection status remains unknown.

Once infected, cattle experience a period of bacteremia that can persist for up to a week [8]. Nonmaintenance pathogenic *Leptospira* spp., such as the serogroup Grippotyphosa [9, 10], cause incidental infections in cattle and are recognized as a leading cause of reproductive failure, icteric abortions, stillbirth, economic loss, and occasionally, meningitis and death [8, 11]. In addition, cattle are well known as maintenance hosts for serogroup Sejroe (mainly serovar Hardjo). The acute phase of such infection is mainly subclinical and often goes unobserved, except in lactating cattle, which might develop agalactia [8]. However, chronic infection associated with the Hardjo bovine-adapted serovar can also lead to reproductive failure, stillbirth, and perinatal death [12, 13]. Regardless of the infection type, cattle may shed pathogenic bacteria in their urine—for up to 40 weeks for Hardjo infection [14]—that can expose humans to the bacteria either directly during the milking process or indirectly following exposure to urine-contaminated water or soil [8, 15, 16].

*Leptospira* infection can be screened through several different routes (blood, serum, urine, and renal tissue) in which *Leptospira* or leptospiral antibodies can be detected [17]. In cattle, blood and serum are typically the most easily accessible, compared to the sampling of urine on farms or renal tissue in slaughterhouses [18, 19], and both blood and serum can be used to perform several leptospiral diagnostic techniques [20]. However, *Leptospira* and/or leptospiral antibody detection can be hindered by differences between the two phases (septicemic and immune) experienced by the host, which leads to an underestimation of its impact [21, 22]. The diagnosis of pathogenic organisms such as *Leptospira* increasingly relies on molecular methods based on the polymerase chain reaction (PCR) [23], which can accurately detect *Leptospira*-derived DNA in the early stages of infection [20, 24, 25]. The serology-based microscopic agglutination test (MAT) is less advantageous for early diagnosis since it only detects antibodies from a past or current infection. The results of the latter test are reliable at the level of the serogroup but cannot be used to detect infectious serovars [26]. In addition, MAT results are interpreted subjectively, which can lead to repeatability issues and variability in the identification of the putative infecting serogroup [27, 28]. Despite these drawbacks, MAT is considered the immunological reference method for the experimental diagnosis of leptospirosis by the World Organization for Animal Health (WOAH) [29] and the World Health Organization (WHO) [30], and is recommended by the former for use in herd *Leptospira* screening [31]. Overall, a complementary approach that combines both PCR and MAT is the best option for improving early detection of biphasic leptospirosis [32].

Within the larger region of the Middle East, attempts have been made to survey cattle for *Leptospira* infection [33]. Prevalence ranged from 0% to 43%, and seroprevalence ranged from 0% to 85%, with Sejroe and Grippotyphosa as the predominant pathogenic serogroups in seropositive cattle. With the exception of a single publication from 1947 [4], though, no epidemiological studies have been carried out in Lebanon to describe the occurrence of *Leptospira* and the risk linked to infection. To fill this gap, this preliminary study aimed to provide an initial characterization of the threat posed by *Leptospira* infection in cattle in Lebanon. Specifically, our goal was to describe the *Leptospira* infection status and circulating serogroups within cattle herds in Lebanon, starting with the governorate of Mount Lebanon (ML), which is an important operational area for raising cattle [34]. Furthermore, we investigated the occurrence of *Leptospira* in imported cattle upon their arrival to Lebanese soil, with the goal of potentially differentiating between the genetic profiles of autochthonous and exotic *Leptospira* DNA.

## 2. Materials and Methods

### 2.1. Provision of Blood Samples

Cattle blood collected during regular professional consultations by a commercial veterinary company—that provides services related to animal importation and development—for their private annual prophylaxis program was used later on in this study for further investigation of *Leptospira* infection in Lebanon. Nonetheless, all herd owners were contacted by the company and agreed to provide cattle specimens for leptospirosis research purposes.

### 2.2. Description of Cattle of Interest

Cattle, particularly of the Holstein breed, are raised in abundance in Lebanon for dairy milk production [35]. The veterinary company that provided blood samples is dedicated to the development of cattle production in this country and therefore imports breeding dairy cattle from Europe, mainly France and Germany. It conducts consultations on dairy herds in all of Lebanon's governorates, but the majority of inspections are carried out in ML, where there are 50 dairy herds of interest to the company. The cattle in the ML herds are mainly of the Holstein breed, and their numbers range between 5 and 80 heads per farm. The leptospirosis vaccine had not been administered to local cattle.

### 2.3. Sampling Design

This study was carried out in Lebanon, in the ML and Beirut governorates (port of Beirut), for local and imported cattle, respectively. Animals were conveniently selected, regardless of their reproductive performance or clinical picture, from 14 of the 50 farms followed by the veterinary company in the ML governorate; this was carried out during the end of the dry season in 2021, in the months of October and November. This amount of sampling would allow the detection of at least one seropositive herd given a minimum between-herd seroprevalence of 20% and an uncertainty of 5% [36]. In addition, a one-time sampling campaign was performed by the company in the same period on a subset of a group of approximately 400 imported cattle upon their arrival at the port of Beirut. Tests were performed at an individual level, however, the serological results were interpreted at the herd level, as recommended by the WOAH manual for terrestrial animals [29]. In order to obtain relevant information at the herd level, samples were provided from 10% of the imported and local herd populations, with a minimum of 10 heads per farm or the whole herd if the total was less than 10, and a 10-cow sample being appropriate to reveal the presence or absence of an infection in a herd [29].

### 2.4. Repartition of Blood Samples

A total of 187 blood and 135 serum samples were provided from cattle. Of the 187 blood specimens, 135 were from arbitrarily chosen animals in herds located in the governorate of ML, while the remaining 52 were from imported livestock. Serum specimens (*n* = 135) were acquired solely from cattle in the ML governorate; they could not be collected from imported livestock due to the stress experienced by the animals, resulting in serum hemolysis following centrifugation. No animal showed clinical signs of illness.

### 2.5. *Leptospira* Microagglutination Testing

Microscopic agglutination tests were performed based on the standard methodology [29] using a panel of live leptospires. In total, twelve *Leptospira* serogroups, with related serovars in parentheses, were used: Australis (Bratislava, australis, munchen), Autumnalis (autumnalis, bim), Ballum (Castellonis), Bataviae (bataviae), Canicola (canicola), Grippotyphosa (grippotyphosa, vanderheidon), Icterohaemorrhagiae (icterohaemorrhagiae, copenhageni), Panama (panama, mangus), Pomona (pomona, mozdok), Pyrogenes (pyrogenes), Sejroe (sejroe, saxkoebing, hardjo, and wolffii), and Tarassovi (tarassovi). Information on the serogroups, serovars, and strains used is available in Table 1. To avoid biases in interpretation, all MAT reactions were analyzed by a single technician. As recommended in the WOAH manual, a 1 : 100 titer was used as the cut-off point for seropositive samples [29]. MAT results were interpreted at a global level, and the epidemiological unit considered was the herd. A herd was considered currently or recently infected at the herd level when at least one animal showed a positive MAT result. However, given the high specificity of MAT, serum samples were primarily tested using a 1 : 50 titer as evidence of previous exposure to *Leptospira*, as suggested by WOAH [29]. Seropositive reactions were analyzed as follows:

1. If a serum specimen demonstrated reactivity to only one serogroup, that serogroup was designated dominant.
2. If a serum specimen reacted to two or more serogroups, but with a difference of threefold or more between the highest and the next highest titer, the former was designated the dominant serogroup.
3. If a serum specimen reacted to two or more serogroups with less than a threefold difference between the highest and the next highest titer, the serogroups were designated equally dominant. This most often occurs as a result of cross reactions [37, 38], and the result was considered inconclusive in this case.

## 3. Molecular Detection of *Leptospira* DNA

### 3.1. DNA Extraction and Purification

DNA was extracted from cattle whole blood samples using the Quick-DNA Miniprep Kit, Cat. No. D4068 (Zymo Research, USA), following the manufacturer's instructions for liquid tissues. In addition, the OneStep PCR Inhibitor Removal Kit, Cat. No. D6030 (50 spin columns/purifications) (Zymo Research, USA), was used directly on the extracted DNA following the manufacturer's instructions, in order to efficiently remove contaminants that might inhibit downstream PCR reactions.

### 3.2. Real-Time PCR Targeting the 16S rRNA Gene

As an initial step, the efficiency of DNA extraction and the absence of inhibitors were tested for each sample by the amplification of the *β*-actin endogenous housekeeping gene. The *β*-actin primers were ^5′^CAGCACAATGAAGATCAAGATCATC^3′^(forward) and ^5′^CGGACTCATCGTACTCCTGCTT^3′^ (reverse), as described in Toussaint et al. [39], and the sequence of the *β*-actin probe was ^5′^^FAM^TCGCTGTCCACCTTCCAGCAGATGT^TAMRA 3′^. Real-time PCR reactions were performed on a Stratagene-Agilent Mx3000P qPCR system.

*β*-actin gene expression also served as an internal control for expression of the 16S rRNA target gene. This gene sequence was amplified from all purified DNA using AgPath-IDOne-StepReal-Time PCR Reagents (Applied Biosystems). Real-Time PCR reactions contained 12.5 *μ*L 2X Real-Time PCR Buffer, 2.5 *μ*L of probe and each primer, 1 *μ*L of 25X Real-Time PCR Enzyme Mix, and 4 *μ*L of DNA in a final volume of 25 *μ*L. Amplification was performed using primers targeting a region of the *Leptospira* rrs (16S) gene that were designed in a previous study, with a slight modification of the cycling protocol [40]. The 16Spatho primers were ^5′^CGGGAGGCAGCAGTTAAGAA^3′^ (forward) and ^5′^AACAACGCTTGCACCATACG^3′^ (reverse). The sequence of the 16Spatho probe was ^5′FAM^GCAATGTGATGATGGTACCTGCCT^BHQ1 3′^, as described in Waggoner et al. [40]. Real-Time PCR cycling was performed on a Stratagene-Agilent Mx3000P qPCR system using the following parameters: 95°C for 10 min, followed by 40 cycles of (1) 95°C for 15 s and (2) 60°C for 1 min. Fluorescence was provided by TaqMan probes (based on reporter and quencher fluorochromes) which continuously detected and reported DNA amplification; a *C*_T_ was automatically set for each DNA sample, and any exponential curve that reached a *C*_T_ prior to cycle 40 was considered a positive result [41]. A no-template mix and a positive control were added in each run of the real-time PCR.

### 3.3. Conventional PCR Targeting leptA and leptB Primers

For samples with positive results in the real-time PCR, conventional PCR (cPCR) was performed with HotStarTaq DNA polymerase (250 U), using primers targeting the *Leptospira* rrs (16S) gene that were designed in previous studies, with a slight modification of the cycling protocol [42]. cPCR reaction mixes contained 5 *μ*L 10x PCR Buffer, 2 *μ*L MgCl_2_ (25 mM), 1 *μ*L leptA primer (10 *μ*M), 1 *μ*L leptB primer (10 *μ*M), 1 *μ*L of dNTPs (10 mM), 0.5 *μ*L Taq polymerase (5 *μ*/*μ*l), 34.5 *μ*L nuclease-free water, and 5 *μ*L of DNA in a final volume of 50 *μ*L. The leptA forward primer (^5′^GGCGGCGCGTCTTAAACATG^3′^) and leptB reverse primer (^5′^TTCCCCCCATTGAGCAAGATT^3′^), specific for the genus *Leptospira*, were used, as described in a previous study [42]. cPCR cycling was performed on an Eppendorf Mastercycler Nexus Gradient Thermal Cycler as follows: 95°C for 15 min, 40 cycles of (1) 95°C for 15 s, (2) 57°C for 30 s, and (3) 72°C for 1 min, and a final elongation step at 72°C for 10 min. A no-template mix and a positive control were added in each run of the cPCR. PCR products were confirmed in duplicate using 1% gel electrophoresis for 30 min at 100 volts, and the size of the amplified DNA fragment was checked under ultraviolet light.

### 3.4. Sanger Sequencing

Samples that were cPCR-amplified and visualized on a 1% agarose gel under ultraviolet light were then Sanger sequenced by a service provider (Genoscreen, Lille, France) using the same primers employed in the cPCR. ChromasPro (version 2.6.6) was used to assemble nucleotide sequences that were at least 330 bp in length and compatible with the genus *Leptospira*. Each contig was queried using the nucleotide Basic Local Alignment Search Tool (BLAST) in the NCBI database (https://blast.ncbi.nlm.nih.gov/) to determine *Leptospira* species assignment. A phylogenetic tree was then generated based on the partial 16S gene rDNA sequences obtained from our blood sample amplicons and reference *Leptospira* DNA sequences provided by the “Laboratoire des Leptospires” [42]. The tree was constructed using Muscle version 5 [43] with IQ-TREE 2.2.0.3 [44], using the maximum likelihood method (log-likelihood −991.593) and the best-fit model TPM3 + G4, chosen based on values of the Bayesian information criterion (BIC). A bootstrap analysis was performed with 1000 replicates (S1 Fig.).

### 3.5. Agreement between PCR and MAT

Compliance between PCR and MAT results at the herd level was determined using Cohen's Kappa coefficient, calculated using Rstudio (version 1.3.1093, “Apricot Nasturtium”) with the formula K=Pr(*a*) − Pr(*e*)/1 − Pr(*e*), where Pr (a) is the observed percentage of agreement and Pr (e) the expected percentage of agreement. This comparison was repeated for two different MAT dilutions: PCR and MAT (titer 1 : 50) and PCR and MAT (titer 1 : 100).

## 4. Results

### 4.1. Molecular Analysis

**Figure 1:**
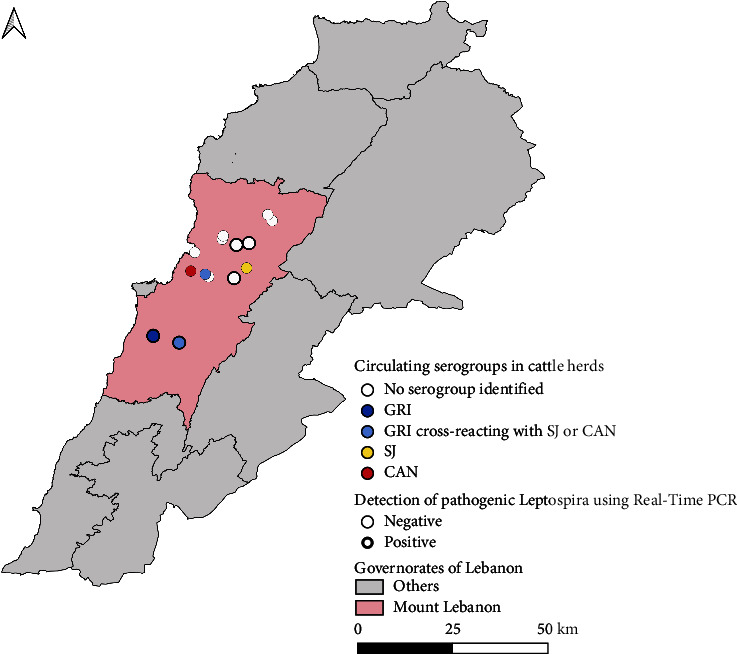
Geographical distribution of circulating serogroups and real-time PCR-positive herds in Mount Lebanon. Map created with DIVA-GIS version 7.5 and designed with QGIS 3.16.14 hannover. GRI, Grippotyphosa; SJ, Sejroe; CAN, Canicola.

*β*-actin gene expression was reported by Real-Time PCR from all 187 DNA samples that were extracted from whole blood. The 16S rRNA gene sequence of *Leptospira* was detected by Real-Time PCR in 7 of 135 local cattle (representing 5 of the 14 herds) and in 1 of 52 imported cattle. For six of the seven Real-TimePCR-positive local animals (representing the same five herds) and the Real-TimePCR-positive imported animal, cPCR amplification revealed a 330-bp fragment compatible with the genus *Leptospira* [45]. All seven sequences demonstrated 100% nucleotide affinity with a published sequence corresponding to *Leptospira kirschneri* (GenBank accession number MK726123.1). The five PCR-positive herds were geographically distributed as follows: two were located in the north, one in central ML, and two in the south (Figure 1).

### 4.2. *Leptospira* Microagglutination Testing in Cattle in Mount Lebanon

Five herds (three in the center and two in the south of ML) contained cattle with MAT titers ≥1 : 100 (Figure 1). Herds *F*, *J*, and *M* each contained a single serogroup—Sejroe (1 : 400), Canicola (1 : 100), and Grippotyphosa (1 : 200), respectively—even though the herds had multiple seropositive animals. The remaining seropositive herds—herds N and G—contained two serogroups each, with neither appearing to be dominant as the reported MAT titers—CAN (1 : 200) and GRI (1 : 200) in herd G, and GRI (1 : 200) and SJ (1 : 100) in herd N—had less than a threefold difference. In herd G, a single seropositive individual demonstrated equal antibody titers against serogroups Canicola and Grippotyphosa, hindering the identification of a single dominant serogroup. In herd N, the two seropositive animals had distinct MAT profiles, with Grippotyphosa as the putative serogroup for the first and Sejroe for the second. For each seropositive animal, the reactive antibody titers for each tested serovar/serogroup in MAT are displayed in Table 2.

### 4.3. Combining Molecular and Serological Results Among Cattle Herds in Mount Lebanon

When we examined both PCR results and MAT results at titers ≥1 : 100, evidence for *Leptospira* or antileptospira antibodies was found in 8 of the 14 tested herds. Cohen's Kappa coefficient was 0.07 with a *p* value of 0.8. When we examined both PCR results and MAT results at titers ≥1 : 50, the total number of positive herds remained the same (8 of 14), but the agreement between the two methods changed. The resulting Cohen's Kappa coefficient was 0.43, with a *p* value of 0.06. The serogroups and species of *Leptospira* found in each herd are presented in Table 2 and Figure 1.

## 5. Discussion

To the best of our knowledge, this is the first study to use molecular and serological methods to characterize bovine *Leptospira* infection in Lebanon, particularly in Mont Lebanon (ML). It also applied the same molecular approach to evaluate *Leptospira* infection among imported cattle upon their arrival to Lebanese soil (port of Beirut) prior to their distribution to Lebanese herds. The identification of pathogenic *Leptospira* species and circulating serogroups in cattle highlights an unaddressed threat to public health in Lebanon, particularly for people working with cattle. In addition, the detection of pathogenic *Leptospira* in imported cattle suggests that animal importation may be one of the means by which pathogenic bacteria like *Leptospira* are introduced to this country.

Pathogenic *L. kirschneri* was the only species detected in all positive cattle, local or imported. This species is known worldwide as an agent of human leptospirosis [16, 46, 47] and can have notable clinical manifestations in some patients, as reported in France [48] and Malaysia [49]. Furthermore, it has been suggested that infection by *L. kirschneri* can also have an impact on herd production output, but the clinical manifestations of such infection are ambiguous [50]. *L. kirschneri* has not been described in studies conducted in neighboring countries, which to date have been limited to reports of molecular positivity in cattle and have not identified the infecting bacterial species. However, this species has been described in cattle in countries in North and South America (Brazil, Uruguay, and Mexico) [50–52]. Our finding of *L. kirschneri* in local herds is consistent with our serological finding of the predominant serogroup Grippotyphosa since the related serovars Grippotyphosa and Vanderheiden, tested in our study, belong to the *L. kirschneri* species [53]. In addition, the fact that *L. kirschneri* was also identified among imported cattle likely destined for introduction into Lebanese herds suggests the potential introduction or maintenance of pathogenic *Leptospira* through global trade.

The number of *Leptospira* infections detected in our study is more likely an underestimation of the genuine occurrence in this population due to the time of sampling as well as the clinical specimen chosen for analysis. The illness has a distinct seasonal pattern and is closely associated with climatic factors [54]. In subtropical countries such as Lebanon, most cases of leptospirosis in both humans and cattle naturally occur following rainfall and flooding [55–58]. In general, flooding is thought to help *Leptospira* disseminate in the environment [59], resulting in leptospirosis transmission and infection, as it has been described in cattle in other subtropical countries [57, 60]. In our study, sampling was performed in October and November, a period with relatively little rainfall, which might have reduced our chances of detecting *Leptospira* through Real-Time PCR. In addition, *Leptospira* bacteria are found in relatively low numbers in the bloodstream, which can impede the identification of infected cattle despite the sensitivity of the 16S Real-Time PCR (7.0 to 2.0 log_10_ copies/*μ*L) [40] and the optimization of primer sequences and annealing temperatures [61]. Moreover, leptospires can only be found in blood during the first week of illness in both humans and animals, mainly from the second to the fourth day of infection [8, 15, 62]. This likely explains the low number of *Leptospira*-infected cattle detected here as well as in two studies conducted in Egypt (country with similar meteorological circumstances and herd management techniques) that also used blood as a sampling matrix [63, 64] and detected *Leptospira* DNA in seven out of 625 cattle blood samples in one study, solely [64]. The use of sample specimens other than cattle blood, such as urine, where leptospires are retrieved for a longer period of time, could have led to a higher incidence report, as obtained in other studies that tested cattle urine and blood samples by Real-Time PCR and only detected *Leptospira* DNA in urine samples [65]. In the population used in our study, whole blood was the only available sample that remained appropriate for bacteremia detection and *Leptospira* characterization, although the detection period was limited to the acute phase of infection. Overall, despite its drawbacks, our approach enabled us to identify *Leptospira* and reveal its presence in domestic cattle with ML, but it was not inappropriate to assess the true prevalence of *Leptospira* infection.

A low degree of concordance between PCR and MAT was observed using a cut-off titer of ≥1 : 100. All cattle which tested positive by Real-Time PCR still had not produced antibodies and were categorized as negative by MAT. The latter finding supports the biphasic nature of leptospirosis, as seroconversion mainly occurs 10 to 14 days following infection [8]. However, some cases of (*Leptospira* serovar Hardjo) infected cattle who survived the bacteremic phase but did not develop agglutinating antibody titers above the 1 : 100 threshold have also been documented in the literature [66, 67]. Still, our work is consistent with previous studies demonstrating that the use of Real-Time PCR in conjunction with MAT increases the sensitivity of *Leptospira* detection, mainly in the early stages of infection [32, 68–70]. Here, the use of both molecular and serological methods within the same herd enabled us to acquire more information about the status of *Leptospira* infection in cattle herds from ML than either method could have revealed by itself.

Although the methodology used most likely underestimates the occurrence of *Leptospira* infection in cattle, it provides some initial data, namely, that *Leptospira kirschneri* and serogroup Grippotyphosa are the predominant species and serogroup, respectively. These results are useful in developing further research studies, therefore, we make the following recommendations. Despite its weakness regarding sensitivity, the methodology used in this paper could be extended in space and time to assess the relative leptospiral risk throughout the remaining governorates of Lebanon and across seasons, assuming that in each case the degree of underestimation would be relatively constant. To improve the precision of prevalence estimates and thus assessments of the level of risk for people in contact with cattle, the sensitivity of detection should be improved by sampling specimens other than blood, such as kidneys (in abattoirs) or urine (in farms and abattoirs), where leptospires persist longer [71].

In Lebanon, cattle carriers of pathogenic *L. kirschneri* can spread the bacteria through their urine and potentially act as a reservoir for humans—particularly farmers in close proximity—and domestic and wild animals [72], as well as a source of water and soil contamination in which the bacteria can remain viable for months in optimal condition [73]. Cattle being an interface between wildlife and humans, managing *Leptospira* infection in cattle herds (e.g., by following an appropriate vaccination plan) can not only reduce the cattle's health impact but also prevent leptospirosis in humans and other animals besides cattle. The finding of *L. kirschneri* in cattle in ML raises a One Health concern for leptospirosis control in Lebanon in order to sustainably ensure the health of the ecosystem, including humans and animals [74]. Consequently, there is a need to implement a response according to the quadripartite One Health concept definition [75] that includes intersectorial mobilization and communication related to the presence of *L. kirschneri* and leptospirosis risk management among veterinarians, farmers, general physicians, and workers in wild mammal associations practicing in Mont Lebanon. In addition, services related to zoonosis management at the ministries of agriculture, public health, and environment should be aware of the leptospirosis risk and be able to support future efforts on intersectorial and collaborative epidemiological surveillance, disease control, and research [76].

One of the findings of this study is the detection of pathogenic *Leptospira* in imported cattle, which highlights the risk related to importation. WOAH recommends the application of a reference test to 10% of each batch of imported cattle [29]. This step cannot ensure a disease-free herd, but it can minimize the potential risk of infection to herds in the importing country. Another approach to improving the sensitivity of the detection of infected herds could be the use of simultaneous direct and indirect detection, but further studies are necessary to assess the efficiency of such a measure.

## 6. Conclusion

Pathogenic *Leptospira* was detected in both local and imported cattle in Lebanon. As a result, leptospirosis risk should be addressed as a public and animal health concern in this country, raising the need to follow a “One Health” approach. Enhancing public awareness is essential, particularly among veterinarians and general physicians, so they can detect and report clinical forms of leptospirosis and consequently, maximize the health of humans, and animals. Additional studies on *Leptospira*-infected populations (e.g., rodents and dogs) should be conducted in Lebanon in order to characterize potential maintenance hosts and have a more thorough understanding of leptospirosis epidemiology to design effective disease control strategies.

## Figures and Tables

**Table 1:** List of the reference strains employed in the MAT antigen panel.

Serogroup	Serovar	Strain
Australis	Muenchen	München C 90
Australis	Australis	Ballico
Australis	Bratislava	Jez Bratislava
Autumnalis	Autumnalis	Akiyami A
Autumnalis	Bim	1051
Ballum	Castellonis	Castellòn 3
Bataviae	Bataviae	Van Tienen
Canicola	Canicola	Hond Utrecht IV
Grippotyphosa	Grippotyphosa	Moskva V
Grippotyphosa	Vanderheiden	Kipod 179
Icterohaemorrhagiae	Icterohaemorrhagiae	ENVN
Icterohaemorrhagiae	Copenhageni	M 20
Panama	Panama	CZ 214K
Panama	Mangus	TRVL/CAREC 137774
Pomona	Pomona	Pomona
Pomona	Mozdok	5621
Pyrogenes	Pyrogenes	Salinem
Sejroe	Sejroe	M 84
Sejroe	Saxkoebing	Mus 24
Sejroe	Wolffii	3705
Sejroe	Hardjo	Hardjoprajitno
Tarassovi	Tarassovi	Perepelitsin

**Table 2:** Serovars and species of *Leptospira* identified in seroreacting and PCR-positive cattle, respectively.

		Distribution of antibody titers among serum samples reacting for each tested serovar in MAT									16S PCR
Herds ID (number of individual cattle tested for each herd)	ID of cattle testing positive by MAT and/or PCR	AUT	CAN	COP	ICT	GRI	SJ	SAX	HJ	WOLF	*Leptospira* spp. identified by sequencing

A (10)	—										—

B (10)	—										—

C (10)	C1	50									—
	C2										*L. kirschneri*
	C3										NP^*∗*^

D (10)	D1										*L. kirschneri*
	D2										*L. kirschneri*

E (10)	E1	50									*L. kirschneri*

F (10)	F1					50					—
	F2						100	400			—

G (10)	G1		200			200					—

H (10)	—										—

I (10)	—										—

J (10)	J1		100								—
	J2				50						—

K (10)	—										—

L (5)	—										—

M (10)	M1					200					—
	M2		50								*L. kirschneri*
	M3					50					—
	M4						50				—
	M5			50	50						—
	M6					200					—

N (10)	N1					200					—
	N2							50			—
	N3						100	100	50	50	—
	N4										*L. kirschneri*

PORT (52)	P1	NP	NP	NP	NP	NP	NP	NP	NP	NP	*L. kirschneri*

AUT, autumnalis; CAN, canicola; COP, copenhageni; ICT, icterohaemorrhagiae; GRI, grippotyphosa; SJ, sejroe; SAX, saxkoebing; HJ, hardjo; WOLF, wolffii; NP, not performed; NP^*∗*^, refers to animals for which real-time PCR was positive but cPCR was negative and therefore sanger sequencing was not performed; P1, refers to imported cattle for which only molecular tests (real-time PCR 16S and cPCR leptoA and B) were performed.

## Data Availability

The data used to support the findings of this study are available from the corresponding author upon request.

## Supplementary Materials

Supplementary Materials S1 Fig. Phylogenetic tree based on partial 16S rDNA gene sequence obtained from blood samples amplicons. The phylogenetic tree was constructed using Muscle version 5 with IQ-TREE 2.2.0.3 using the maximum likelihood method and the best-fit model TPM3 + G4. A bootstrap analysis of 1000 replicates was performed. Samples colored in red correspond to the *L. kirschneri* cluster.
