# Supplementary figures and images for: Molecular and Serological Identification of Pathogenic Leptospira in Local and Imported Cattle from Lebanon
Source: Transbound Emerg Dis. 2023 Feb 27;2023:3784416. doi: 10.1155/2023/3784416 (PMC12017195; doi:10.1155/2023/3784416)

Tree scale: 0.1

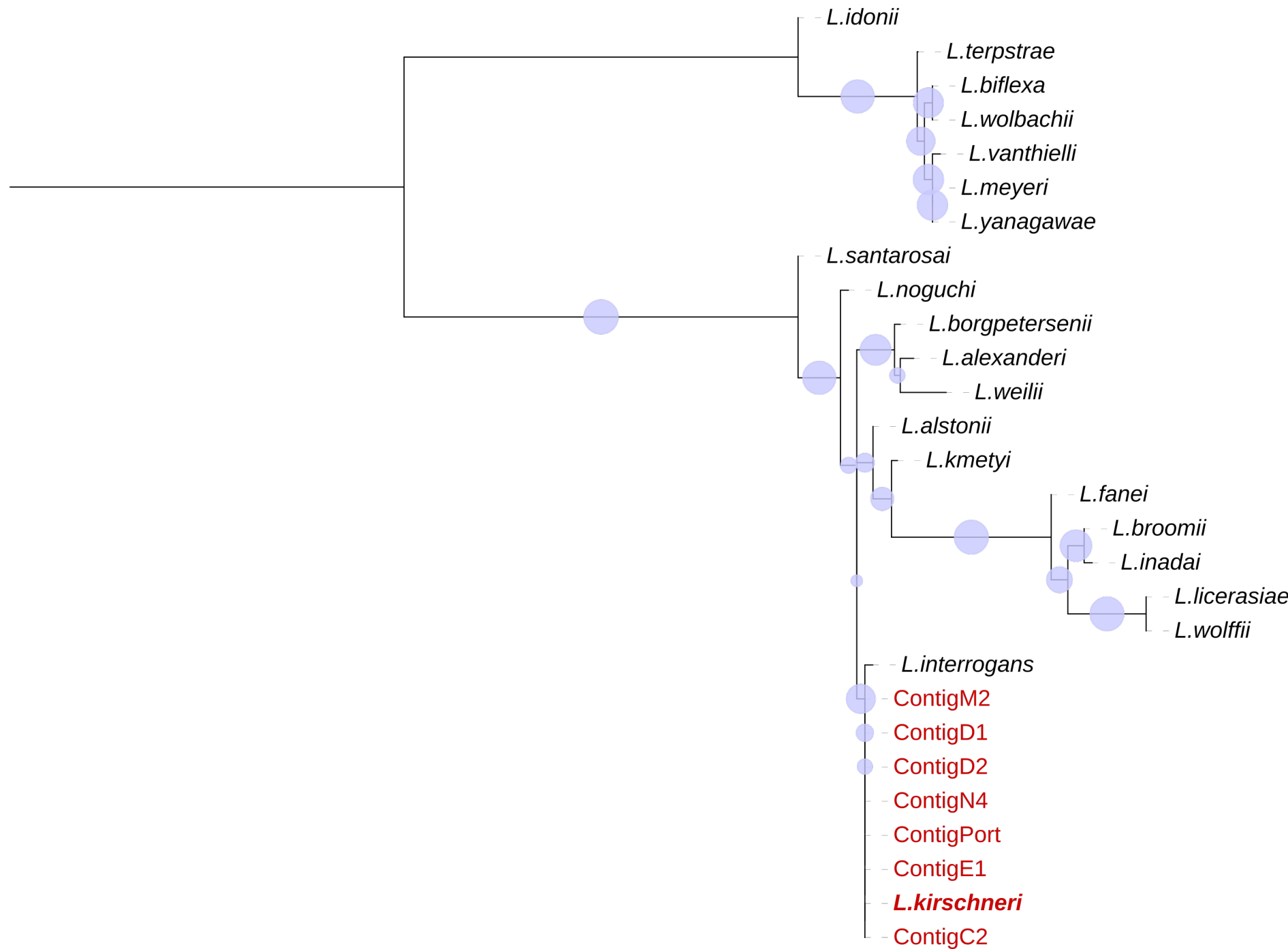

Supplement: Supplementary Materials — S1 Fig. Phylogenetic tree based on partial 16S rDNA gene sequence obtained from blood samples amplicons. The phylogenetic tree was constructed using Muscle version 5 with IQ-TREE 2.2.0.3 using the maximum likelihood method and the best-fit model TPM3 + G4. A bootstrap analysis of 1000 replicates was performed. Samples colored in red correspond to the L. kirschneri cluster. [file 3784416.f1.pdf]
